# Screen, Sample, Stratify: Biomarkers and Machine Learning Compress Dementia Pathways

**DOI:** 10.3390/biomedicines14010159

**Published:** 2026-01-12

**Authors:** Simone Battaglia, Masaru Tanaka

**Affiliations:** 1 Faculty of Psychology, eCampus University, 22060 Novedrate, Italy; simone.battaglia@uniecampus.it; 2 Department of Psychology, University of Turin, 10124 Turin, Italy; 3 Danube Neuroscience Research Laboratory, HUN-REN-SZTE Neuroscience Research Group, Hungarian Research Network, University of Szeged (HUN-REN-SZTE), H-6725 Szeged, Hungary

## 1. Vignette and Pressure Point

Late-life cognitive complaints seldom align with a single disease entity. Common neurodegenerative pathologies explain only a portion of decline, leaving substantial variance unresolved [1]. Biological aging processes—cellular senescence, inflammation, and mitochondrial dysfunction—form a shared substrate for cognitive vulnerability [2]. Depression further complicates attribution by accelerating biological aging, increasing dementia risk, and altering trajectories of attention, memory, and executive control [3,4,5,6,7]. Neuropsychiatric symptoms are frequent in mild cognitive impairment (MCI) and can precede progressive neurodegeneration [8]. Structural magnetic resonance imaging (MRI) highlights frontal and temporal atrophy with white matter injury in late-life depression [8]. Comparative imaging and genomic syntheses reveal convergence with MCI, preserving diagnostic ambiguity at first presentation [9].

At presentation, the differential is precarious. Dementia with Lewy bodies (DLB), Alzheimer’s disease (AD), and Parkinson’s disease dementia (PDD) share terrain yet diverge in attentional and visuospatial profiles, motor signs, and biomarkers, complicating early diagnosis [3,10,11]. Continuum models show heavier cerebral amyloid angiopathy, tau in DLB, and faster tempo than PDD, linking proteinopathy to vascular load [1,2]. Quantitative electroencephalography (EEG) and pragmatic prodromal algorithms improve separation from AD [12]. In multiple sclerosis (MS), fatigue, depression, and pain converge through shared inflammatory and network-level mechanisms, further blurring clinical attribution [4].

Nervous system disorders are now the leading cause of disability, with neurodegeneration driving escalating economic demand [1,4]. Uncertainty multiplies costs: impaired financial decision-making, caregiver strain, and post-stroke cognitive sequelae increase service use and dependency [1,2]. Pandemic disruptions and psychiatric cognitive impairment amplify system load, even when decline remains subjective [5,9]. This editorial adopts an integrative format to contextualize and connect the contributions of this Special Issue, highlighting shared mechanisms, diagnostic bottlenecks, and translational opportunities rather than providing an exhaustive review.

## 2. From Nosology to Mechanism: Rewiring Clinical Decisions

Guidelines have improved baseline practice, yet failure modes remain visible at precisely the points that matter, particularly in MCI, where heterogeneity and shifting thresholds produce discrepant classifications, high false negatives, and unstable diagnoses [13,14]. These instabilities dilute trials, defer counseling, and blur care pathways. Post-stroke cognitive impairment guidance still relies on thin evidence for prognosis and treatment, limiting timely intervention [15]. Prognostic factors such as anticholinergic burden are inconsistent, yielding low-certainty recommendations and weak individual prediction [16]. In major depressive disorder, cognitive dysfunction often persists despite symptomatic remission, while assessment remains uneven and understandardized, reducing actionable targets [16]. Latency to effective care also reflects adherence barriers in bipolar disorder, where multifactorial nonadherence undermines otherwise sound protocols [17]. Methodological upgrades are promising, including deep learning models for response prediction that could shorten decision cycles, but deployment requires calibration, transparency, and prospective validation [18,19,20].

Rewiring clinical decisions begins with shifting from static phenotypic labels to dynamic, mechanism-informed risk models. Transdiagnostic frameworks emphasize shared circuitry, trajectories, and prediction rather than category boundaries [21,22,23]. Precision medicine operationalizes this shift through multi-omics, biomarker panels, and data-driven stratification that personalize care pathways [16,24,25]. Mechanistic signal can be captured with EEG and machine learning for early diagnosis and prognosis in MCI and AD [4], complemented by digital phenotyping that yields explainable, longitudinal profiles [14,26]. Plasma biomarkers refine individual risk in prodromal states [27]. Network-based classifications and integrated neuroimaging–genetics approaches parse heterogeneity and nominate actionable subtypes in mood disorders and depression [28,29].

## 3. The Translational Corridor: Detection → Anchoring → Intervention

This corridor links rapid case finding to mechanism and then to action. We start with bedside triage and scalable blood biomarkers, where p tau217 and brief cognition guide rule-in or rule-out [30]. Visual queries in Lewy body disease (LBD), EEG signals, and fluorodeoxyglucose positron emission tomography (FDG-PET) sharpen early differentiation [31]. We then anchor risk in circuits and systems biology to define targets. Finally, calibrated models and task-coupled neuromodulation inform matched interventions and real clinic workflows.

### 3.1. Detection and Stratification

Early detection benefits from precise bedside triage. In this Special Issue, Balz et al. validate a revised Tracking Assessment of Cognition in Multiple Sclerosis (TRACK-MS) battery with improved sensitivity and specificity for cognitive impairment in multiple sclerosis, enabling rapid case finding and targeted referral [32]. Complementing this, Capogna et al. synthesize evidence on minor visual phenomena (MVP) in LBD, positioning brief visual queries as discriminative signals that precede fuller syndromic expression [33] (Table 1). Nuanced phenotyping remains essential given frequent Alzheimer’s co-pathology in LBD [34]. Short tools such as the ALBA instrument extend screening to nonspecialist settings, while EEG markers capture posterior network dysfunction that supports early differentiation [35,36]. When accessible, FDG-PET increases diagnostic accuracy over clinical assessment alone [37]. Translational LBD models underscore why phenotype-first gatekeeping accelerates the corridor from detection to intervention [38].

Along the corridor, rule-in and rule-out decisions increasingly rely on scalable blood biomarkers paired with genetic risk stratification [51,52]. Lai et al. synthesize convergent evidence that plasma phosphorylated tau 217 (p-tau217) leads Alzheimer’s pathology, with strong PET and cerebrospinal fluid (CSF) correlations and high accuracy on automated platforms across primary and secondary care [39]. Performance generalizes to Asian and South American cohorts, and a two-cutoff strategy improves clinical triage in subjective decline and MCI [53,54,55,56,57]. In parallel, Bao et al. present a clinical model that predicts the carriage of pathogenic or likely pathogenic variants in dementia with a positive family history, enabling targeted sequencing and counseling [40] (Table 1). Triggering receptor expressed on myeloid cells 2 (TREM2) risk and presenilin 1 (PSEN1) location shape trajectories and trial design [58,59]. Together, these tools reduce time to mechanism-informed care.

Algorithmic risk integration in Parkinson’s disease (PD) is moving from proof of concept to actionable triage. Mohammadi et al. report high discriminative performance using readily obtainable clinical variables and blood biomarkers, demonstrating that pragmatic Random Forest pipelines can flag early cognitive decline for intensified monitoring [41] (Table 1). Broader evidence shows that multimodal models that fuse clinical, CSF, imaging, and genetic features sharpen prediction and reveal the relevance of Alzheimer’s co-pathology [60,61,62,63]. Deep architectures improve sequential forecasting and event timing, from temporal models to disease progression frameworks [64,65]. Modality-specific pipelines add leverage: FDG-PET, sleep EEG, and dopamine transporter single photon emission computed tomography (DAT-SPECT) with cognitive scales enhance conversion prediction [66,67]. Meta-analyses underscore robust sensitivity and specificity across modalities, while highlighting the need for calibration, drift surveillance, and external validation before routine deployment [68,69].

Signals at the eye–brain interface justify targeted cognitive screening. Vision impairment is associated with decline, and glaucoma elevates dementia risk across subtypes [70,71]. Exfoliation glaucoma shows greater cognitive burden, with non-invasive biomarkers such as advanced glycation end products (AGEs) and carotenoids suggesting triage avenues [72,73]. Kadoh et al. demonstrate that patients with exfoliation glaucoma exhibit significantly lower Mini-Cog scores and higher fingertip AGE levels than those with primary open-angle glaucoma, highlighting AGE accumulation as a potential mechanistic and diagnostic bridge between ocular and cognitive dysfunction [42] (Table 1). Screen older adults with glaucoma, prioritizing normal tension, severe, or rapidly progressive disease, and those with optical coherence tomography angiography (OCTA) vessel density changes. Genetic and cortical links to AD strengthen timing for routine checks in this population [74,75].

A pragmatic triad is emerging for routine practice: rapid cognitive screening, blood panel, and composite risk trajectory. Plasma p-tau, amyloid beta 42 to 40 ratio (Aβ42/40), neurofilament light chain (NfL), and glial fibrillary acidic protein (GFAP) enable scalable rule-in and risk prediction across preclinical and prodromal states [76,77]. Two-step workflows that deploy p-tau217 first, reserving confirmatory tests for uncertain cases, reduce invasive procedures while preserving accuracy [78]. Composite models that fuse p-tau217 with brief cognition outperform single markers for MCI progression, and amyloid tau neurodegeneration framework (ATN) profiles plus demographics refine forecasts in unimpaired elders [77,79]. Outside AD, pairing blood NfL with MRI improves MS triage; trajectory clustering personalizes follow-up [80].

Generalizability hinges on context. Multicenter studies reveal site effects and training set bias that distort risk estimates unless explicitly modeled [81,82]. Demographic calibration is mandatory, as age, sex, ancestry, and education reweight signals across modalities [83,84]. Performance is stage contingent; tau-PET, MRI volumetry, EEG, and molecular imaging show shifting accuracy from preclinical states to MCI and dementia [85,86].

### 3.2. Mechanistic Anchors

Biondi et al. map structural alterations that track mood-related deficits in schizophrenia, sharpening the link between anatomy and affective symptomatology [43] (Table 1). Converging evidence positions endophenotypes as the translational bridge: they tie genetic risk to circuit dysfunction and clinical expression [87,88]. Thalamic reticular abnormalities offer a mechanistic anchor for attentional and mood disturbances, while large-scale network hypo-connectivity in high-risk states reinforces system-level vulnerability [89]. Electrophysiological signatures align with these structural findings and retain heritable signal [90]. Therapeutically, circuit-informed strategies matter: dorsolateral prefrontal stimulation reduces depressive symptoms, and emerging agents target GABAergic and glutamatergic nodes. Together, these anchors convert descriptive mood comorbidity into measurable, modifiable neural targets [91].

Plasticity and vascular integrity sit at the core of mechanism-first care [92]. Statsenko et al. distill hallmarks of brain plasticity into an operational scaffold that spans synaptic remodeling, glial–neuronal crosstalk, metabolic control, and structural adaptability, aligning with broader frameworks of neurodegenerative hallmarks and systems models of neurons, glia, extracellular matrix, and the neurovascular unit [44]. Complementing this axis, de Lima et al. map a triad that couples vascular impairment with muscle atrophy and cognitive decline, motivating integrative prevention and rehabilitation strategies [45] (Table 1). Blood–brain barrier breakdown emerges early and independently predicts decline, while neurovascular uncoupling implicates astrocytic and endothelial dysfunction in aging and AD [93,94]. Endothelial caveolae calibrate neurovascular coupling and energy delivery [95]. Translationally, inflammatory and blood–brain barrier (BBB) biomarkers anchor vascular cognitive impairment pipelines with clear clinical hooks [96].

Depression occupies multiple positions along the neurodegenerative arc: antecedent risk, prodromal signal, and consequence of progressive pathology. Papa et al. synthesize bidirectional, stage-dependent links and argue for longitudinal designs that disentangle trajectories and mechanisms [46] (Table 1). Converging evidence shows that late-life depression heightens conversion from subjective or mild impairment to dementia, with neuropsychiatric features sharpening risk stratification [97,98]. Biological pathways span stress hormones, cytokine signaling, and impaired neurogenesis that interface with Alzheimer pathophysiology [99,100,101]. Mild behavioral impairment further elevates risk, aligning affective dysregulation with early network disruption [102,103,104].

Longitudinal readouts should be coupled with circuit probes and vascular–muscle indices with plasticity markers, while transcranial magnetic stimulation (TMS) excitability and resting-state EEG track decline [105,106]. Hippocampal atrophy, arterial spin labeling magnetic resonance imaging (ASL MRI) or ultrasound metrics, and plasma panels, including NfL, GFAP, sphingosine 1 phosphate (S1P), and osteopontin, forecast progression across subjective cognitive decline (SCD), PD, and AD [107,108,109,110,111,112].

### 3.3. Intervention and Delivery

Intrathecal delivery is emerging as a pivotal route for mechanism-addressed neurotherapeutics in neurodegeneration [113]. Schreiner et al. synthesize the clinical rationale, showing how bypassing the BBB can accelerate target engagement while exposing persistent delivery gaps [47] (Table 1). Indications span proteins, antisense oligonucleotides, and gene therapies requiring reliable CSF exposure and parenchymal penetration [114]. Distribution is constrained by CSF dynamics, meningeal interfaces, and molecular size, demanding optimized physicochemistry and dosing schedules [115]. Device innovation is pressing: engineered catheters, controlled-release systems, and nanoporous implants enable intrathecal pseudodelivery and sustained gradients [113]. Nanomedicine and targeted nanoparticles promise better retention and tissue targeting yet face pharmacokinetic hurdles [116]. Quantitative, model-based design should guide trials and patient selection [117].

Targeted neuromodulation is converging on mechanism-guided, task-coupled protocols for post-ischemic cognitive recovery [118]. Markowska et al. map molecular alterations in the ischemic brain to stimulation-responsive pathways and advocate parameterized protocols with rational combination therapies [48] (Table 1). Meta-analyses show dorsolateral prefrontal tDCS and TMS improve cognition, with effects shaped by montage, dose, and task coupling [119,120]. Mechanistic bridges include the modulation of functional connectivity, synaptic plasticity, and brain-derived neurotrophic factor (BDNF), with convergent enhancements of long-term potentiation (LTP) and N-methyl-D-aspartate receptor (NMDAR) signaling in animal models [121]. Task-embedded designs matter: tDCS reshapes EEG during working memory, normalizes age-altered functional magnetic resonance imaging (fMRI) networks, and tunes functional near-infrared spectroscopy (fNIRS) hemodynamics [122]. Dual-site high-definition transcranial direct current stimulation (HD-tDCS) with TMS-EEG sharpens circuit-specific targeting [123].

Urological physiology is not peripheral to MS care—it interlocks with fatigue and mood outcomes. Jaekel et al. show that defined urodynamic abnormalities increase fatigue risk, motivating longitudinal assessment and targeted management [49] (Table 1). Symptom network analyses connect fatigue to specific depressive items and to urgency and voided volume, nominating shared intervention nodes [124]. Converging mechanisms include neuroinflammation and disrupted reward processing [124]. Care pathways should integrate bladder-focused therapy with exercise and mood treatment, supported by registry and longitudinal data showing bidirectional fatigue–depression effects and persistent undertreatment [49,124].

Peripheral proxy biomarkers attract clinical interest yet demand rigor. Carpita et al. report lower intra-platelet BDNF in adults with autism linked to symptom burden, underscoring age and methodological effects [50]. Meta-analyses show elevated peripheral BDNF, but heterogeneity and matrix differences persist (Table 1). Platelet count and preanalytical factors confound serum signals. Technical reviews emphasize assay standardization and replication before clinical translation [125].

## 4. Operationalization—Bottlenecks, Upgrades, Redesign, Practice Capsule, and Closeout

Operationalization turns intention into action across the corridor. We start by confronting credibility bottlenecks: p-tau217 assay standardization with transportable cutoffs; strict model calibration; cross-site robustness; alignment of patient reported outcomes (PROs) with mechanisms; and equitable access. Next come near term upgrades with explicit metrics, then a longer horizon redesign that uses endotype-stratified platform trials and regulatory grade qualification. A concise practice capsule guides use now versus caution, while the closeout codifies transportable thresholds and locked analyses [126,127].

### 4.1. Bottlenecks to Credibility

Bottlenecks to credibility begin at the bench. Plasma p-tau217 shows strong diagnostic promise, yet credibility depends on assay harmonization and transportable cutoffs that survive lot-to-lot drift, platform differences, and demographic shifts [128]. Reviews of p-tau217 and related epitopes argue for multi-cohort calibration anchored to CSF comparators and pathology, with predefined decision thresholds and proficiency testing to guard against silent misclassification [129]. In parallel, predictive models require rigorous calibration, not only high discrimination. Large neuroimaging and genetic benchmarks show that apparent accuracy collapses without external validation, well-specified calibration curves, and pre-registered analytic plans [130]. Thousands of individuals are typically needed to stabilize brain–behavior associations; smaller studies should preferentially report uncertainty, sensitivity to nuisance variance, and domain shift. Reproducible pipelines and site-robust components, as exemplified by automated independent component analysis (ICA) frameworks and multi-site bipolar MRI challenges, help contain scanner effects and analytic degrees of freedom, but they are not substitutes for cross-site replication with locked code and fixed thresholds.

Credibility also hinges on aligning what matters to patients with what models and biomarkers claim to measure. Work on neuropsychiatric outcomes after chimeric antigen receptor T cell therapy (CAR-T) and validated attribution algorithms in systemic autoimmune disease illustrate how carefully constructed patient-reported outcomes can be tied to mechanistic hypotheses and adjudicated endpoints. Embedding such PROs as co-primary readouts with p-tau217 and imaging markers can reveal when signals are clinically meaningful versus statistically convenient. Finally, equity is a methodological requirement. Dementia research and neurologic care remain unevenly distributed across regions and racial or ethnic groups. Without inclusive sampling frames, shared standards for data and assay exchange, and cost-sensitive deployment plans, calibrated models and cutoffs will amplify disparities rather than close them. Credibility therefore emerges from a linked agenda: standardize assays against common references, calibrate and externally validate models at scale, make analyses more robust against site effects, align PROs to mechanisms, and design access pathways that are fair by construction [131].

### 4.2. Near-Term Upgrades (12–24 mo) with Hard Metrics

A pragmatic path forward is a two-step workflow that separates case finding from confirmation. For Parkinson’s and multiple system atrophy (MSA), a first-stage digital or multimodal screen achieves high discrimination in external cohorts, with area under the curve (AUC) typically 0.85 to 0.94, and in some settings higher [132]. Adding a confirmatory, pathogenesis-based biomarker in step two tightens specificity and stabilizes net reclassification improvement (NRI) compared with usual care that relies on clinical judgment alone [133]. Proteomic and exosomal signatures, including neuronal and oligodendroglial α-synuclein assays, deliver validation sensitivities around 90 percent with comparable specificity, while proteomic panels refined in longitudinal registries push step two AUC toward 0.98. Parallel refinements in dementia triage matter for differential diagnosis. Protocolized screening for MVP combined with a structured cingulate island sign rating on FDG-PET reduces Lewy body misclassification, cuts false positives against AD, and prevents overcalling atypical parkinsonism when visual symptoms are the earliest clue.

Credibility then hinges on prospective calibration across at least three sites for both PD machine learning models and p-tau217. Targets should be explicit—area under the receiver operating characteristic curve (AUROC) at or above 0.75 with a calibration slope close to 1.0, intercept near 0, and decision curve net benefit that exceeds usual care across plausible thresholds. Achieving this requires a minimal multimodal panel that can survive real clinical constraints. A blood anchor such as p-tau217 or p-tau181, a brief cognitive composite, and a lightweight digital measure captured passively or in short tasks. Predefine acceptable operational metrics—missingness under 5 percent across sites, interclass correlation at or above 0.8 for repeated measures, cross cohort drift assessed quarterly, and lockbox external validation before any update. Evidence from wearables, metabolomics, and proteomics shows that heterogeneous signals outperform self-report and lifestyle alone, but only when device firmware, preprocessing, and batch correction are versioned and shared. With that discipline, the screen to confirm paradigm becomes an implementable service line rather than a single site curiosity.

### 4.3. Long-Horizon Redesign (3–5+ yrs)

A credible long-horizon redesign should center on endotype-stratified platform trials that co-test pharmacologic and device strategies in one adaptive architecture [134]. Intrathecal agents can be assigned to proteinopathy-enriched strata while non-invasive brain stimulation (NIBS) arms are personalized by network topology, symptom domain, and target engagement [135]. Entry, allocation, and graduation rules should be gated by regulatory-grade readouts. Plasma p-tau217, with validated cutoffs and longitudinal responsiveness, functions as a selection and monitoring surrogate, while digital phenotypes from brief cognitive panels and passively acquired behavior provide high-frequency safety and efficacy signals suitable for interim decisions. This pairing enables efficient enrichment, early futility stopping, and standardized evidence packages for regulators.

Prevention must shift to mechanism-first programs along the vascular–muscle–cognition axis by embedding exercise and nutrition interventions as default backbones in at-risk cohorts, anchoring endpoints to neural physiology, not only global cognition, and combining p-tau217 or related plasma markers with vascular metrics, sarcopenia indices, and brief digital cognition to link mechanistic engagement to clinical change. Multidomain trials already demonstrate that structured activity, diet, vascular risk control, and cognitive training yield measurable neural benefits; next-generation studies should hard-wire these components as shared control or background therapy across arms, with standardized target-engagement milestones and upgradeable intensity.

The final pillar is an interoperable data commons that stitches preclinical tasks to clinical readouts. Common data elements, FAIR metadata, and harmonized pipelines allow rodent or nonhuman primate assays of memory, motor control, and network plasticity to map onto human digital and imaging endpoints. Advanced analytics, including topological and representation learning, can preserve cross-species geometry while exposing mechanistic signatures that are invariant to platform and site. With this scaffold, signals discovered in models translate into prospectively testable biomarkers and outcomes, and negative findings retire non-performing mechanisms early rather than after years of costly drift.

### 4.4. Practice Capsule

TRACK-MS offers a rapid, validated cognitive tracking option for MS that rivals longer batteries while fitting real clinic constraints [136]. Structured visual queries for LBD, centered on the cingulate island sign rating on FDG-PET, improve diagnostic specificity and reduce miscalls against AD when applied with a brief symptom checklist. Context-validated p-tau217 on fully automated platforms now supports referral, triage, and longitudinal monitoring; analytical performance and cross-site accuracy meet the threshold for regulatory-grade decision support when paired with predefined cutoffs and periodic proficiency testing.

Use with caution: platelet BDNF fluctuates with platelet release dynamics, mood state, storage conditions, and comorbidity, which limits specificity for neurodegenerative indications; treat as a secondary signal. Site-specific machine learning without external calibration invites optimistic bias; require at least one independent cohort, calibration slope near 1.0, and net benefit beyond usual care before deployment. Watchlist: intrathecal delivery platforms remain promising for concentrated target engagement yet need safety gating, pharmacokinetic-pharmacodynamic bridging, and mechanistic futility stops. Combined NIBS plus pharmacology protocols merit structured exploration with endotype stratification, dose titration anchored to target engagement, and harmonized outcomes that capture both symptom change and network-level physiology [137].

### 4.5. Close—“The Corridor Contract”

One sentence delta: starting today, multicenter calibration is the default gatekeeper for any biomarker, imaging readout, or machine learning tool that moves beyond discovery into practice. The Alzheimer’s Disease Neuroimaging Initiative (ADNI) showed how shared protocols and open repositories convert variability into signal, while Enhancing NeuroImaging Genetics through Meta-Analysis (ENIGMA) and related consortia proved that credible neuropsychiatry now depends on cross-site integration at scale. Harmonized MRI and PET methods, common data elements, and prospective proficiency testing have matured to the point that single site claims are insufficient. Multicenter validation of blood biomarkers such as neurofilament light underscores both feasibility and necessity, and reviews across modalities converge on the same conclusion.

Join a shared platform trial that couples endotype stratification with a standing calibration core. Contribute data under common elements, run locked analyses with preregistered decision thresholds, and adopt site readiness checks before enrollment. Imaging will follow harmonized acquisition and phantom procedures. Blood assays will use standardized materials and quarterly drift audits. Digital and ML endpoints will require external calibration curves and independent cohorts. The corridor is not merely a metaphor but a commitment to calibration over novelty, transportability over isolated performance, and integration over silos.

## Figures and Tables

**Table 1:** Corridors for early dementia pathways. This table organizes the Special Issue contributions along three translational corridors—Detection and Stratification, Mechanistic Anchors, and Intervention and Delivery—highlighting how each compresses the path from diagnosis to mechanism-informed care.

Corridor	Sub-domain	Study/Paper (Short)	Population/Context	Modality/Approach	Primary Contribution	Ref.
3.1. Detection and Stratification	Phenotypic triage instruments	TRACK-MS bedside screen	MS	Brief cognitive battery	Higher sensitivity/specificity for early MS cognitive impairment → rapid case finding/referral	[32]
		LBD minor visual phenomena (MVP)	LBD	Symptom questionnaire/synthesis	Validates brief visual queries as early discriminants preceding syndromic expression	[33]
	Biomarker rule-in/rule-out	Plasma p-tau217	AD (primary/secondary care)	Blood biomarker (automated platforms)	Strong CSF/PET correlations; two-cutoff triage improves accuracy and scalability	[39]
		Variant-carrier dementia model	Familial dementia clinic	Clinical risk model for genetics	Predicts carriage of pathogenic/likely pathogenic variants to target sequencing/counseling	[40]
	Algorithmic risk integration	ML for PD cognitive decline	PD	RF using clinical + blood markers	High discrimination for early decline; pragmatic pipeline for intensified monitoring	[41]
	Cross-domain signals	Glaucoma–cognition interface	POAG and exfoliation glaucoma	Neuro-ophthalmic/cognitive profiling	Identifies glaucoma-linked cognitive burden and screening “who/when” cues	[42]
3.2. Mechanistic Anchors	Circuit-level endophenotypes	Mood-linked deficits in SCZ	SCZ	Structural MRI, systems/circuit readouts	Maps anatomy tied to affective symptoms; endophenotype bridge to mechanisms	[43]
	Systems biology axes	Hallmarks of brain plasticity	Cross-diagnostic	Conceptual/mechanistic synthesis	Operational scaffold of plasticity (synaptic, glial–neuronal, metabolic, structural)	[44]
		Vascular–muscle–cognition coupling	Aging/cognitive decline	Vascular and sarcopenia metrics	Triad linking vascular impairment, muscle atrophy, and cognitive loss; clinical hooks	[45]
	Affective staging continuum	Depression vs. neurodegeneration	Late-life and prodromal states	Scoping synthesis	Positions depression as risk/prodrome/consequence; emphasizes longitudinal designs	[46]
3.3. Intervention and Delivery	Route-of-delivery innovation	Intrathecal neurotherapeutics	Neurodegeneration	CSF delivery science/devices	Defines indications, CSF dynamics limits, and device/nanomedicine gaps	[47]
	Targeted neuromodulation mapping	TMS/tDCS × molecular change × tasks	Post-ischemic cognitive recovery	NIBS with task coupling	Parameterized, mechanism-guided protocols; connectivity/plasticity bridges	[48]
	Symptom network → target translation	MS urodynamics → fatigue/depression care	MS	Urodynamics + symptom networks	Urodynamic abnormalities predict fatigue; integrates bladder, exercise, mood care	[49]
	Peripheral proxy biomarkers	Platelet BDNF in ASD	Autism (adults)	Platelet/serum BDNF	Links intra-platelet BDNF to symptom burden; flags assay/processing pitfalls	[50]

AD, Alzheimer’s disease; ASD, autism spectrum disorder; BDNF, brain-derived neurotrophic factor; CSF, cerebrospinal fluid; LBD, Lewy body disease; ML, machine learning; MRI, magnetic resonance imaging; MS, multiple sclerosis; MVP, minor visual phenomena; NIBS, non-invasive brain stimulation; PD, Parkinson’s disease; PET, positron emission tomography; POAG, primary open-angle glaucoma; p-tau217, phosphorylated tau at threonine 217; RF, random forest; SCZ, schizophrenia; tDCS, transcranial direct current stimulation; TMS, transcranial magnetic stimulation; TRACK-MS, Tracking Assessment of Cognition in Multiple Sclerosis.

## Abbreviations

The following abbreviations are used in this manuscript:

AD	Alzheimer’s disease
ADNI	Alzheimer’s Disease Neuroimaging Initiative
AGEs	advanced glycation end products
ASD	autism spectrum disorder
ASL MRI	arterial spin labeling magnetic resonance imaging
ATN	amyloid tau neurodegeneration framework
AUC	area under the curve
AUROC	area under the receiver operating characteristic curve
Aβ42/40	amyloid beta 42 to 40 ratio
BBB	blood–brain barrier
BDNF	brain-derived neurotrophic factor
CAR-T	chimeric antigen receptor T cell therapy
CSF	cerebrospinal fluid
DAT-SPECT	dopamine transporter single photon emission computed tomography
DLB	dementia with Lewy bodies
EEG	electroencephalography
ENIGMA	Enhancing NeuroImaging Genetics through Meta-Analysis
FDG-PET	fluorodeoxyglucose positron emission tomography
fMRI	functional magnetic resonance imaging
fNIRS	functional near-infrared spectroscopy
GFAP	glial fibrillary acidic protein
HD-tDCS	high-definition transcranial direct current stimulation
ICA	independent component analysis
LBD	Lewy body disease
LTP	long-term potentiation
MCI	mild cognitive impairment
MRI	magnetic resonance imaging
MS	multiple sclerosis
MSA	multiple system atrophy
MVP	minor visual phenomena
NfL	neurofilament light chain
NIBS	non-invasive brain stimulation
NMDAR	N-methyl-D-aspartate receptor
NRI	net reclassification improvement
OCTA	optical coherence tomography angiography
p-tau217	phosphorylated tau at threonine 217
PD	Parkinson’s disease
PDD	Parkinson’s disease dementia
PET	positron emission tomography
POAG	primary open-angle glaucoma
PROs	patient reported outcomes
PSEN1	presenilin 1
RF	random forest
S1P	sphingosine 1 phosphate
SCD	subjective cognitive decline
SCZ	schizophrenia
TRACK-MS	Tracking Assessment of Cognition in Multiple Sclerosis
tDCS	transcranial direct current stimulation
TMS	transcranial magnetic stimulation
TREM2	triggering receptor expressed on myeloid cells 2
